# Potential Nociceptive Regulatory Effect of Probiotic* Lactobacillus rhamnosus* PB01 (DSM 14870) on Mechanical Sensitivity in Diet-Induced Obesity Model

**DOI:** 10.1155/2016/5080438

**Published:** 2016-08-25

**Authors:** Fereshteh Dardmeh, Hans Ingolf Nielsen, Hiva Alipour, Benedict Kjærgaard, Erik Brandsborg, Parisa Gazerani

**Affiliations:** ^1^Biomedicine Group, Department of Health Science and Technology, Faculty of Medicine, Aalborg University, Aalborg, Denmark; ^2^SMI®, Department of Health Science and Technology, Faculty of Medicine, Aalborg University, Aalborg, Denmark; ^3^Department of Clinical Medicine, The Faculty of Medicine, Aalborg University Hospital, Aalborg, Denmark; ^4^Bifodan A/S, Hundested, Denmark

## Abstract

Treatments for obesity have been shown to reduce pain secondary to weight loss. Intestinal microbiota, as an endogenous factor, influences obesity and pain sensitivity but the effect of oral probiotic supplementation on musculoskeletal pain perception has not been studied systematically. The present study examined the effect of a single daily oral dose (1 × 10^9^ CFU) of probiotics (*Lactobacillus rhamnosus* PB01, DSM14870) supplement on mechanical pain thresholds in behaving diet-induced obese (DIO) mice and their normal weight (NW) controls. The mice (*N* = 24, 6-week-old male) were randomly divided into four groups on either standard or high fat diet with and without probiotic supplementation. Both DIO and NW groups with probiotic supplementation maintained an insignificant weight gain while the control groups gained significant weight (*P* < 0.05). Similarly, both DIO and NW probiotics supplemented groups demonstrated a significantly (*P* < 0.05) lower sensitivity to mechanical stimulation compared to their corresponding control. The results of this study suggest a protective effect of probiotics on nociception circuits, which propose a direct result of the weight reduction or an indirect result of anti-inflammatory properties of the probiotics. Deciphering the exact underlying mechanism of the weight loss and lowering nociception effect of the probiotic applied in this study require further investigation.

## 1. Introduction

Physiological pain plays a life-essential protective role, while acute or chronic pathological pain indicates a medical problem that needs treatment and imposes a medical challenge [1].

Pain perception is a complex process including neuronal, nonneuronal, and cognitive processes modulated by peripheral and central factors. Neurotransmitters, immune cells, and hormones have been demonstrated to contribute in pathogenesis of chronic pain [2]. Several biological and psychological factors may interfere with perception of pain. To mention a few, gender, depression, or genetic factors have been shown to alter pain perception both in humans and animals [3–5].

Since pain is a subjective concept and pain measurement is challenging, it is usually measured under controlled conditions, by responses to experimental stimuli as applied by mechanical, thermal, electrical, and chemical stimuli [6].

Pressure pain threshold (PPT) has been identified as a reliable and easy biomarker in multiple clinical pain states [7, 8]. Rodent withdrawal reflex to pressure application upon sensation of pain is interpreted as similar to PPT assessments in humans.

Pain threshold is influenced by several factors, including obesity, which alters adipose tissue metabolic and endocrine functions leading to alterations in systemic physiology including an increased release of fatty acids, hormones, and proinflammatory molecules that contribute to obesity associated complications [9]. Several studies have shown that pain sensation or threshold to painful stimuli can change due to a state of obesity but controversial results exist [10–14]. Some clinical studies have demonstrated a higher pressure pain threshold in obese compared to nonobese humans patients [11, 14]. Other studies have demonstrated that obese humans and rats are more sensitive to pain stimuli than normal weighted ones [15, 16]. Controversies might be due to several factors, including ignorance of confounding variables such as gender, age, socioeconomic factors, and comorbidities such as diabetes, hypertension and chronic pain, methodological differences, type of stimuli, sex, and age. In development of obesity, intestinal microbiota has been considered as an essential factor [5].

Previous studies have demonstrated a relationship between intestinal microbiota and diseases including pain disorders [17–20] with probiotics having a positive effect.

The possible beneficial role of probiotic supplementation on management of chronic musculoskeletal pain is in its infancy and requires further investigation. To study the confounding factors under well-controlled conditions, experimental models in animals and humans are superior to patient studies. In particular, rodent behavioral models have been important tools for advancing our understanding of the physiology underlying nociception and pain [3]. Diet-induced obese (DIO) models in mice can assist in investigations deciphering the etiology of human obesity since they closely mimic the result of the high fat/high density foods in modern societies during the past decades [21].

In this study we seek to address if probiotic supplements can potentially overcome the deleterious effects of obesity and reduce pain sensitivity by assessing the effect of* Lactobacillus rhamnosus* (PB01, DSM 14870) supplement on PPT in DIO mice. The results of this study would potentially prove beneficial in treatment strategies and predictive algorithm in conditions with existence of both obesity and pain.

## 2. Materials and Methods

Animal experiments in this study were carried out in accordance with the Guidelines for Animal Experimentation and Approval of “The Danish Animal Experiments Inspectorate” number 2014-15-0201-00026.

### 2.1. Animals

Twenty-four, 6-week-old male C57BL/6NTac mice were acquired (Taconic, Denmark) and housed in a room at 22°C to 24°C, with 60% relative humidity and 12 h dark-light cycles (light on from 0800 to 2000 h). Mice were allowed two weeks of adaptation and free access to their respective diets and tap water ad libitum during the study period.

### 2.2. Study Design

*Phase I*. Following adaptation, the mice were randomly divided into two groups and fed on standard diet as normal weight (NW) models while the other group were fed on an industry-standard high fat (60%) research rodent diet (D12492, Research Diets Inc., USA) to create the diet-induced obesity (DIO) models.

*Phase II*. Four weeks after feeding on the standard or fat diet, each group was divided into two subgroups continuing the previous diet with or without probiotic supplements creating the following four diet groups: normal diet (ND); normal diet + probiotic supplement (NDPR); fat diet (FD); and fat diet + probiotic supplement (FDPR).

### 2.3. Probiotics

*Lactobacillus rhamnosus* PB01, DSM 14870, was provided in the form of Lyophilized powder by Bifodan A/S (Hundested, Denmark). Based on the product information from the manufacturer (Bifodan A/S, Copenhagen, Denmark), the strain PB01 had been genetically characterized employing two biomolecular techniques. Species-level identification, achieved by means of 16S rRNA gene sequencing, demonstrated that PB01 belonged to the species* Lactobacillus rhamnosus.* Pulsed Field Gel Electrophoresis (PFGE), employed for strain typing, enabled obtaining a strain specific macrorestriction pattern. Previous studies have reported the positive effect of L.* rhamnosus* PB01 vaginal administration (ECOVAG®) in the treatment of vaginosis [22]. The same strain was offered to us by Bifodan A/S (Hundsted, Denmark) and suggested (based on unpublished preliminary studies) to also have positive beneficial effects while administered orally.

Aliquots providing 1 × 10^9^ CFU per mouse were prepared (based on manufactures guidelines) and stored at −20°C until administration. Shortly before use, the prepared probiotic aliquots were diluted in normal saline (0.25 mL per mouse) at room temperature as vehicle and administered orally to the NDPR and FDPR groups.

The ND and FD groups received oral administration of normal saline without probiotics. This process was repeated once every day during phase II (second 4 weeks) of the study. Probiotic administration was performed using a gavage needle to ascertain the presence of the probiotics in the gastrointestinal tract.

### 2.4. Pressure Pain Sensitivity

Sensitivity to mechanical stimulation, as a translational biomarker of pain sensitivity, was assessed by the electronic Von Frey (Bioseb, France). This test evaluates how soon a withdrawal of the paw will occur due to a standard mechanical pressure (pressure pain threshold, PPT, in grams). The more sensitive it is to pain, the faster the withdrawal reaction would be detected. The mice were weighed using a digital scale and the Von Frey test was performed once every two weeks 3 times on all mice. Mice were placed in an acrylic box restrainer (15 × 15 cm) with a metal mesh floor (5 × 5 mm square openings) to prevent extra movement while having enough space to show reaction to mechanical stimulation. A filament (0.1–10 *μ*L pipette tip) attached to the sensor of the Von Frey device was applied with a steadily increasing pressure to the plantar surface of the mouse's hind-paw until withdrawal occurred. The amount of pressure (g) at the time of hind-paw retraction was recorded by the Von Frey device. Averages of three consecutive readings (with 3-minute intervals) from the same hind paw were recorded for further statistical analysis.

### 2.5. ELISA Tests

At baseline and the end of weeks 4 and 8, blood samples were collected from the facial vein of conscious mice [23]. Blood serum was obtained after centrifugation (500 g for 10 min at 4°C) and stored at −20°C until being used to assess blood lipid profiles (total cholesterol (TC), high density lipoprotein (HDL), low/very low density lipoprotein (LDL/VLDL), and cholesterol) using a commercially available ELISA assay kit (ab65390, ABCAM, UK) according to the manufacturer's directions.

### 2.6. Statistical Analysis

Data are shown as means ± standard deviation (SD). Repeated measures ANOVA was used to compare differences in pain sensitivity and weight between groups and over time points. Bonferroni* post hoc* test was used for pair-wise comparison wherever ANOVA yielded a statistical significant difference. Lipid profile parameters (TC, HDL, and LDL/VLDL) were compared by one way ANOVA. A *P* value less than 0.05 (*P* < 0.05) in these analyses was considered significant unless otherwise stated. The “IBM® SPSS® Statistics” version 23 was used to perform the mentioned statistical analysis

## 3. Results

### 3.1. Probiotics Supplementation Effects on Body Weight

As expected, all groups demonstrated a rising trend throughout the study period. However, the FD group, which were on a high fat diet, gained more weight compared to the ND and resulting in a significant weight difference (*P* < 0.01) at week 4.

**Figure 1:**
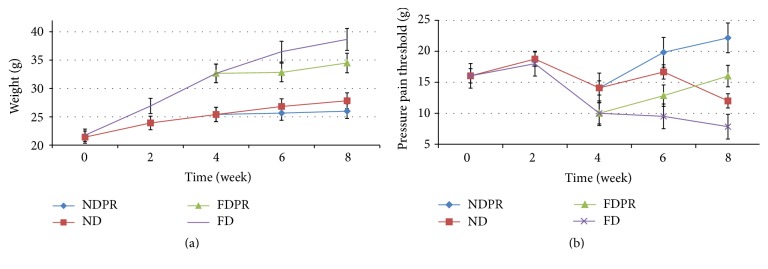
The abilities of* L. rhamnosus* PB01, DSM 14870, to reduce body weight (a) and increase pressure pain threshold (b) in mice fed normal diet (ND), high fat diet (FD), normal diet with probiotics (NDPR), and fat diet with probiotics (FDPR) during 8 weeks. Data are means (*n* = 6) and whiskers indicate SD (standard deviation).

After week 4, both ND and FD groups maintained a rising trend in the weight, whereas the groups which received the probiotic supplement maintained a stable weight with no significant change up to week 8 (Figure 1(a)).

The stable weight of the FDPR and the rising trend in the FD group led to a nonsignificant difference at week 6 (*P* = 0.08) and continued to a significant difference at week 8 (*P* < 0.002).

The NDPR group maintained a stable weight after the start of probiotic supplementation at week 4 whereas the ND group remained stable until week 8 (Figure 1(a)).

### 3.2. Probiotics Supplementation Effects on Pressure Pain Threshold

The results demonstrate striking changes in the pain pressure threshold (PPT) values in both NW and DIO mice after 4 weeks of the probiotic supplementation (Figure 1(b)).

At the start of the study (baseline) PPT ranged between 15 and 17.7 g. A slight increase in the PPT was observed during the first 2 weeks of the study with each group being maintained on their corresponding diet (normal or fat diet) with no significant difference (Figure 1(b)).

However, a clear significant (*P* < 0.01) difference was observed between the groups maintained on normal and fat diet at week 4 (Figure 1(b)).

The FD group continued the decreasing trend in (PPT) until week 8, whereas the FDPR group demonstrated an increasing PPT (lower pain sensitivity) after the start of probiotic supplementation (from week 4 to week 8) reaching a significant (*P* < 0.01) difference in PPT compared to the FD group on week 8 (Figure 1(b)).

The ND group demonstrated a rise in the PPT between weeks 4 and 6 changing to a decreasing trend from weeks 6 to 8 (Figure 1(b)).

The NDPR group also demonstrated an increasing PPT (lower pain sensitivity) after the start of probiotic supplementation (from week 4 to week 8) reaching a significant (*P* < 0.01) difference in PPT compared to the ND group at week 8 (Figure 1(b)).

At the end of this study, week 8, the results of PPT were significantly (*P* < 0.01) different between all groups with the NDPR demonstrating the highest PPT in the FDPR, ND, and FD groups, respectively (22.17, 16, 12, and 7.83 (g)) (Figure 1(b)).

### 3.3. Probiotics Supplementation Effects on Lipid Profiles

The serum lipid profiles (serum total cholesterol, LDL-cholesterol, and HDL-cholesterol) of the mice at the start of the study (base) and after 4 and 8 weeks of the respective diet with or without probiotic supplement are presented in Table 1.

## 4. Discussion and Conclusion

The novelty and main purpose of this study were to investigate the potential of the probiotic* Lactobacillus rhamnosus* supplementation on weight gain and pain sensitivity by assessing pain thresholds in DIO mice versus controls.

### 4.1. Effect of Probiotics on Body Weight

Our results demonstrated that DIO group had a higher body weigh compared with NW. Both of the normal and fat diet groups receiving probiotic supplements maintained a stable weight whereas their corresponding diet group without probiotic supplementation continued to gain weight until the end of study at week 8. This observation supported the report by Sanchez et al. that the probiotics (L.* rhamnosus* CGMCC1.3724) could lower body weight [24].

The gut's microbial community is recognized as one of the particular factors related to obesity and metabolic disorders [25–27]. Many studies have demonstrated various mechanisms of antiobesity of* Lactobacillus*, such as regulation of lipid and glucose metabolism [26, 28], production of conjugated linoleic acid [29, 30], reduction of adipocyte size and increase of numbers of small adipocytes in white adipose tissue [31, 32], and regulation of leptin [33].

### 4.2. Effect of Probiotics on Serum Lipid Profiles

Our study demonstrated that total cholesterol levels and LDL/VLDL levels had a rising trend over time in both normal and fat diet groups. Both of the NW and DIO groups receiving probiotic supplements demonstrated lower total cholesterol and LDL/VLDL levels while their corresponding diet group without probiotic supplements showed higher total cholesterol and LDL/VLDL levels throughout the study. Obesity is generally accompanied by an increased concentration of serum total cholesterol [34]. Numerous mechanisms have been suggested for the hypolipidemic activity of probiotics including “deconjugating bile acids through bile salt hydrolase catalysis, uptake and assimilation of cholesterol for stabilization of the cell membrane and binding cholesterol to cell walls by probiotics in the intestine, conversion of cholesterol into coprostanol, inhibition of hepatic cholesterol synthesis by short chain fatty acids such as propionate produced by probiotic bacteria and/or redistribution of cholesterol from plasma to the liver [35]”. Still, many of these proposed mechanisms and experimental evidence specifically targeting cholesterol-lowering effects of probiotics remain controversial [36].

Many studies have reported the hypolipidemic effect of several probiotics strains in animals which is also observed and in line with the results of this study [37–44].

Several studies in humans also confirm the cholesterol-lowering effects of probiotics [45–47] whereas a few studies have reported no contribution of probiotics to any lipid profile changes [48–51]. The controversial results may be due to differences in strains used and the delivery method where the matrix (as a probiotic carrier) may influence the cholesterol-lowering effect of probiotics [36].

### 4.3. Pressure Pain Threshold in DIO and NW Mice

Our results demonstrated that DIO group had a lower mechanical pain pressure threshold (higher pain sensitivity) compared with groups treated with the normal diet.

Results from this study are in line with the findings of several previous reports generally demonstrating a significantly higher pain sensitivity in obese humans [15, 52] and diabetic obese rats [53].

Obese rats receiving intradermal carrageenan injection in the paw were also shown to exhibit greater peripheral inflammation and hyperalgesia compared to lean rats [54] suggesting an inflammatory response potentiated by obesity or various neurophysiological changes as a result of weight gain contributing to the change in nociceptive processing [55].

It has been reported that obesity share a low grade chronic inflammatory state [56]. Cani et al. also showed that high fat feeding induced a low tone inflammation which originates from the intestinal absorption of the lipopolysaccharide (LPS) [57].

Guilherme et al. have reported that hypertrophy of adipocytes due to increased triglycerides uptake to these cells could result in the induction of a chronic inflammatory state by the recruitment of macrophages within the adipose tissue [58].

de Goeij et al. provide additional evidence that systemic inflammation accompanied by changes in pain perception could result in “inflammation-induced increased pain sensitivity”[59].

Other studies evaluating experimentally induced pain in obese and lean human and animal models in laboratory settings have resulted in somewhat controversial results. Some studies reported increased pain response to thermal noxious stimuli and pressure in obese rats [16, 53] while others show no difference in pain behaviors in response to pressure or thermal stimuli [55] or reduced hyperalgesia response [60].

Earlier studies have demonstrated that obese people exhibited decreased pain threshold to mechanical stimuli [15]. Inconsistent results from several studies across body parts in humans also suggest that the reduced pain threshold in obesity may not be seen in all body areas [55].

### 4.4. Effect of Probiotics on Pressure Pain Threshold

In this study a major increase in pressure pain threshold rates (lower sensitivity) was observed in groups with probiotic consumption compared with the groups with no probiotic supplement. Several lines of research have declared that probiotics exhibit powerful anti-inflammatory properties [61, 62]. Other previous studies have indicated a dependency on the intestinal microflora and on the dysregulated immune response to their antigenic structures for the development of gut associated inflammatory conditions, ranging from allergies to autoimmune and inflammatory diseases [63]. Yokokura [64] also reported increased levels of the inflammatory immune response associated cytokine IL-2, with the oral application of “*L. casei* strain Shirota.” This anti-inflammatory effect could also explain the reduced pain sensitivity in the test groups with probiotic supplementation in this study. However, other unknown mechanisms might be involved.

Regardless of the underlying mechanism, probiotics can be suggested as candidates of a novel strategy for weight and pain control in accordance with the management of pain in cases of obesity or normal weight. Translation of this result in humans can potentially suggest a novel therapeutic strategy in pain management of obese or normal weight individuals.

In conclusion this study demonstrated the potential of* Lactobacillus rhamnosus* (PB01, DSM 14870) probiotic supplementation as a weight and pain sensitivity regulator in diet-induced obesity mice model that could indirectly be associated with the induced weight reduction or anti-inflammatory properties of the probiotic.

## Figures and Tables

**Table 1:** Lipid profiles in mice fed normal diet (ND), high fat diet (FD), normal diet with probiotics (NDPR), and fat diet with probiotics (FDPR) at the start of the study (base) and at 4 and 8 weeks into the study (4 W and 8 W). Provided values are the mean ± standard deviation. Similar superscripted letters demonstrate pairwise significant (*P* < 0.05) differences.

Lipid profile parameters	Base	ND 4 W	ND 8 W	NDPR 8 W	FD 4 W	FD 8 W	FDPR 8 W
LDL/VLDL	0.17 ± 0.03	0.17 ± 0.02	0.18 ± 0.02^b^	0.14 ± 0.02^c^	0.19 ± 0.06	0.32 ± 0.05^a,b^	0.21 ± 0.03^a,c^
HDL	0.44 ± 0.06	0.35 ± 0.05	0.66 ± 0.16^c^	0.56 ± 0.05^a,b^	1.17 ± 0.27	1.42 ± 0.24^b^	1.46 ± 0.46^a,c^
Total cholesterol	0.72 ± 0.08	0.88 ± 0.20	1.05 ± 0.15^a,d^	0.96 ± 0.09^b,c^	1.57 ± 0.22	1.73 ± 0.01^a,b,e^	1.56 ± 0.07^c,d,e^
